# *Candidozyma cisalpinoae* sp. nov., a Genomically Distinct, Flower‐Associated Yeast, Resistant to Azoles and Exhibiting Pathogenicity‐Related Traits

**DOI:** 10.1002/yea.70012

**Published:** 2026-03-05

**Authors:** Anna Paula O. Tironi, Katharina O. Barros, Luiz Felipe A. Santana, Daniela L. Souza, Ana Raquel O. Santos, Giovana R. Ávila, Thiago M. Batista, Glória R. Franco, Raphael S. Pimenta, Paula B. Morais, Marc‐André Lachance, Carlos A. Rosa, Susana Johann

**Affiliations:** ^1^ Departamento de Microbiologia, ICB, C.P. 486 Universidade Federal de Minas Gerais Belo Horizonte Brazil; ^2^ Departamento de Bioquímica e Imunologia, ICB, C.P. 486 Universidade Federal de Minas Gerais Belo Horizonte Brazil; ^3^ Instituto Nacional da Mata Atlântica Santa Teresa Brazil; ^4^ Laboratorio de Microbiologia Ambiental e Biotecnologia Universidade Federal do Tocantins Palmas Brazil; ^5^ Department of Biology University of Western Ontario London Ontario Canada

**Keywords:** antifungal resistant, *Candidozyma cisalpinoae* sp. nov, Cerrado biome, flowers, pathogenicity, resistance factors, thermotolerance, tropical flowers, virulence factors

## Abstract

Six yeast isolates were recovered from *Ipomoea* flowers collected in the Cerrado biome of Tocantins, Brazil. Sequence analyses of the ITS–5.8S region and the D1/D2 domains of the large subunit (LSU) rRNA gene indicated that these isolates represent a novel species of the genus *Candidozyma*, phylogenetically related to *Candidozyma auris* and *Ca. ruelliae*. A phylogenomic analysis based on 2116 single‐copy orthologs from *Candidozyma* species with available whole‐genome sequences showed that the new species, represented by strain UFMG‐CM‐Y6065, is a sister species to *Ca. ruelliae*. The name *Candidozyma cisalpinoae* sp. nov. (MycoBank no. 861366) is proposed to accommodate the new species. The holotype is CBS16108. Sporulation or other evidence of sexual reproduction was not observed, although the genome sequence showed the presence of a functional mating type locus (*MAT*a) and functional pheromone peptides, indicating that the species is haplontic and heterothallic. The species exhibited resistance to multiple antifungals, growth at 42°C, biofilm formation, adhesion to buccal epithelial cells, and expression of efflux pumps, traits of clinical relevance that have been reported for other species in the genus *Candidozyma*.

## Summary

- The novel species *Candidozyma cisalpinoae* was isolated, identified, and formally described.
- Phylogenomic analyses show *Ca. cisalpinoae* is a distinct species closely related to *Ca. ruelliae* within the *Candidozyma* clade.
- *Ca. cisalpinoae* showed resistance to azole antifungals.
- *Ca. cisalpinoae* displays virulence‐associated traits, including thermotolerance, epithelial adhesion, and biofilm formation.
- Rhodamine 6G efflux assays revealed active ABC‐type drug transport, suggesting efflux‐mediated tolerance to azoles.

## Graphical abstract

*Candidozyma cisalpinoae* sp. nov. represents a distinct species within the *Candidozyma* clade, supported by multilocus phylogeny, genome‐scale metrics, and phenotypic traits. Although isolated from floral substrates, this species displays several traits commonly associated with opportunistic pathogenic yeasts.

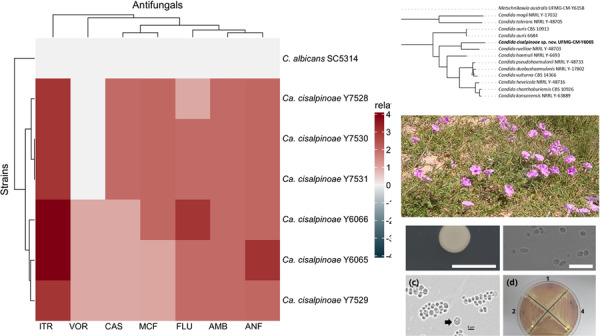

## 1 Introduction

The genus *Candidozyma* was recently proposed by Liu et al. (2024) to accommodate members of the *Candida auris‐C. haemuli* clade within the family Metschnikowiaceae, order Serinales (Groenewald et al. 2023). The genus comprises multidrug‐resistant pathogenic species isolated from both clinical and natural environments. Currently, 13 species have been described, including clinically relevant yeasts such as *Candidozyma auris, Ca. khanbhai, Ca. haemuli, Ca. duobushaemuli, Ca. pseudohaemuli*, and *Ca. vulturna*. The genus also includes species isolated from flowers and tree bark, namely, *Ca. chanthaburiensis, Ca. konsanensis, Ca. heveicola*, *Ca*. *metrosideri*, *Ca. ohialehuae, Ca. ruelliae* (Liu et al. 2024). Recently, *Ca. molenica* was isolated from clinical settings in the Netherlands, and from environmental sources in Belize (Zandijk et al. 2025). *Candidozyma* species form a well‐supported, monophyletic assemblage based on ITS and D1/D2 LSU rRNA analyses, as well as phylogenomic data (Liu et al. 2024; Opulente et al. 2024). The genus harbors clinically important yeasts with resistance to multiple antifungal drugs, which appears to be a unique characteristic compared to other genera in the family *Metschnikowiaceae* (Liu et al. 2024).

Among the members of the genus *Candidozyma* known for their resistance to azole antifungals, *Ca. auris* has notably emerged on multiple continents, exhibiting multidrug resistance across several classes of antifungals (Ciurea et al. 2021). The emergence of *Ca. auris* is thought to be linked to global warming, as it presents high thermotolerance (Casadevall et al. 2021; Cosio et al. 2025). Indeed, *Candidozyma* species can grow at and withstand up to 42°C (Saluja and Prasad 2008; de Jong et al. 2023). Six *Candidozyma* species were either first identified or predominantly isolated in clinical settings. Among them, *Ca. auris* has limited treatment options, high mortality rates, and the ability to spread easily in healthcare settings (Kordalewska and Perlin 2019; Sherry et al. 2017; Gómez‐Gaviria et al. 2023). Furthermore, *Ca. ohialehuae*, an environmental species resistant to echinocandins, has been isolated from flowers (Klaps et al. 2020). Other species, such as *Ca. heveicola* and *Ca. ruelliae*, have been isolated from natural habitats, including flowers and tree bark (Liu et al. 2024). The origin of the opportunistic species is controversial, and for example, *Ca. auris* is theorized to have evolved from a plant saprotroph, possibly becoming a human pathogen after adaptation to higher temperatures (Casadevall et al. 2021). The species has been highlighted as one of four fungal pathogens of critical importance in the recently published World Health Organization (WHO) fungal pathogens priority list in part due to the number of large outbreaks, but also due to the multi‐drug‐resistant nature of the pathogen (Fisher and Denning 2023). Therefore, the discovery of new *Candidozyma* species that have not yet been observed in clinical settings can help understand how these species became opportunistic pathogens, as well as the potential risk they may pose to humans and other animals.

During a survey of flower‐associated yeasts across Brazilian forest biomes, six isolates of a previously unrecognized species of *Candidozyma* were obtained. Analysis of the D1/D2 domains of the LSU rRNA gene, supported by phylogenomic analyses, indicated that these isolates represent a novel species related to *Ca. ruelliae*. The present study provides the formal description of the new species and integrates morphological, physiological and genomic data to define its taxonomic placement. Considering the medical relevance of several members of the genus, we further assessed traits associated with virulence and antifungal response to evaluate the potential clinical significance of the novel species.

## 2 Methods

### 2.1 Yeast Isolation and Identification

The isolates UFMG‐CM‐Y6065, UFMG‐CM‐Y6066, UFMG‐CM‐Y7528, UFMG‐CM‐Y7529, UFMG‐CM‐Y7530 and UFMG‐CM‐Y7531 were recovered from flowers of an unidentified species of *Ipomoea* (Convolvulaceae) collected in the Cerrado ecosystem of the State Ecological Park of Jalapão (10° 38′ 46″ S, 46° 69′49″ W)—state of Tocantins, Brazil, in May 2007. This region is characterized by a semi‐arid tropical climate with high average annual temperatures ranging from 27°C to 30°C. The flowers were collected approximately 50 meters from a secondary road within the park. The nectary region of *Ipomoea* flowers was gently scraped with a sterile loop and inoculated onto YM agar (1.0% glucose, 0.5% peptone, 0.3% malt extract, 0.3% yeast extract, 2.0% agar) containing 100 mg L^‐1^ of chloramphenicol and incubated at room temperature (28°C ± 2°C) for 48 h. Yeast colonies with distinct morphologies were isolated and plated onto the same medium without chloramphenicol for purification and preserved in GYMP broth (2% glucose, 0.5% yeast extract, 0.5% malt extract, 0.2% Na_2_HPO_4_) containing 20% glycerol at ‐80°C. Morphological and physiological characterization was performed using standard methodologies, as described by Kurtzman et al. (2011). The isolates were tested for ascospore production individually or mixed in pairs on GY agar (1% glucose, 0.01% yeast extract, 2% agar), cornmeal, acetate agar (1% glucose, 1.8% potassium chloride, 8.2% sodium acetate trihydrate, 2.5% yeast extract and 1.5% agar) and malt extract agar plates (5% malt extract agar, 2% agar), and incubated at 25°C and 18°C for up to 12 weeks (Kurtzman et al. 2011).

Species identification was performed by sequencing the ITS 5.8 S region (primers ITS1 and ITS4) and the D1/D2 domains of the large subunit rRNA gene (primers NL1 and NL4) (White et al. 1990; O'Donnell 1993; Kurtzman and Robnett 1998; Lachance et al. 1999). The amplified DNA was concentrated, cleaned, and sequenced in an ABI 3130 Genetic Analyzer automated sequencing system (Life Technologies) using BigDye v3.1 and POP7 polymer. The sequences were edited and compared with those in the GenBank database using the Basic Local Alignment Search Tool (Sayers et al. 2021). A Neighbor‐Joining phylogram based on the LSU rRNA gene D1/D2 domain sequences of *Candidozyma* and related species was constructed using MEGA 7 (Kumar et al. 2016). The alignment of 540 positions was generated with the Multiple Alignment using the Fast Fourier Transform (MAFFT) (Katoh and Standley 2016) implementation of the Geneious Prime package, which was also used to identify genes associated with mating. Bootstrap (1000 replicates) values above 50% are shown. The tree was scaled to the number of nucleotide differences between OTUs (Operational Taxonomic Units).

### 2.2 Genome Sequencing, Assembly and Phylogenetic Analysis

For genomic DNA isolation, strain UFMG‐CM‐Y6065 was grown in liquid YM medium at 25°C under agitation. Cell walls were digested with 200 units of Lyticase (Merck, Massachusetts, USA), and genomic DNA was subsequently purified using the Yeast DNA Preparation Kit (Cellco Biotec, São Carlos, Brazil). DNA integrity and concentration were assessed by agarose gel electrophoresis and fluorometric quantification with the Qubit™ dsDNA Assay Kit (Thermo Fisher Scientific, Waltham, MA, USA), respectively. Sequencing libraries were constructed with the NEBNext® Ultra™ DNA Library Prep Kit for Illumina® (New England Biolabs, Ipswich, MA, USA) and sequenced on an Illumina NextSeq. 2000 platform, generating paired end reads of 300 bp.

Raw reads were mapped to the RefSeq Bacterial Database using BWA (Li and Durbin 2009) to filter out potential cross‐contaminants. Unmapped paired‐end reads were then merged into single‐end reads using PEAR v0.9.8 (Zhang et al. 2014). The genome was assembled de novo with SPAdes version 3.15.5 (Prjibelski et al. 2020) with the parameter “‐‐isolate.” Assembly quality was evaluated with the *scaffold_stats.pl* script (https://github.com/sujaikumar/assemblage/tree/master/, last accessed on October 20, 2023) and Quast (Gurevich et al. 2013) version 5.2.0. Repetitive elements were identified with RepeatModeler (Flynn et al. 2020) v2.0.3 and masked in the genome assembly using RepeatMasker (Chen 2004) v4.1.4. Gene prediction was performed using Maker2 (Holt and Yandell 2011) v3.01.04, with Augustus and Snap as gene predictors, and coding sequences were annotated functionally using the Swissprot and TrEMBL databases with Diamond/BlastX (Buchfink et al. 2015). Transfer RNA was detected using tRNAscan‐SE (Lowe and Eddy 1997).

A maximum‐likelihood (ML) phylogenomic tree was reconstructed based on the 2226 single‐copy orthologs identified by BUSCO version 5.5.0 (Manni et al. 2021) which were present in all members of *Candidozyma* with whole genomes available. *Metschnikowia australis* was used as outgroup (all 13 genomes ID used are listed in Supporting Information S1: Table 1). Each BUSCO sequence was aligned using muscle version 3.8.1551 (Edgar 2004), quality‐trimmed with trimAL version 1.2 (Capella‐Gutiérrez et al. 2009) and concatenated to generate a single matrix with 875,243 positions using the BUSCO_phylogenomics script (https://github.com/jamiemcg/BUSCO_phylogenomics, last accessed on April 03, 2024). We inferred an ML phylogeny using iq‐tree version 2.0.7 (Minh et al. 2020), with 1000 ultrafast bootstrap replicates, a partitioning scheme and substitution models. Average nucleotide identity (ANI) values were calculated using orthoANI (Lee et al. 2016). Amino acid Average Identity (AAI) values were calculated using CompareM v0.1.2 (https://github.com/dparks1134/CompareM, last accessed on September 03, 2024) and Percentage of Conserved Proteins (POCP) values were calculated using POCP‐nf (Hölzer 2024).

### 2.3 Determination of Virulence Factors and Antifungal Susceptibility

As the novel species is phylogenetically related to the *Ca. auris–Ca. haemuli* clade, we hypothesized that it could also exhibit antifungal resistance or virulence‐associated traits. The virulence assays performed included antifungal susceptibility testing, biofilm formation, efflux pump activity, and adhesion to human buccal epithelial cells (HBECs). *Candida albicans* SC5314 (kindly provided by Prof. Dr. Eleftherios Mylonakis, Brown University, USA), a well‐characterized clinical strain, was used as control in all assays because it possesses all virulence factors evaluated in this study.

In vitro antifungal susceptibility of the novel species was determined following the broth microdilution method described in the Clinical and Laboratory Standards Institute (CLSI) document M27‐A3 (CLSI 2008). Assays were performed in sterile, flat‐bottom, 96‐well microplates (Difco Laboratories, Detroit, MI, USA). The antifungal agents tested included anidulafungin, micafungin, caspofungin, amphotericin B, and the azoles fluconazole, itraconazole, and voriconazole. Minimum inhibitory concentrations (MICs) were visually determined by turbidity after 24 and 48 h of incubation at 37°C. Wells without drug and wells without yeast cells were included as controls. MIC values were interpreted according to CLSI M27‐A3 guidelines. For comparative visualization, MIC values were transformed to log2 scale and normalized relative to the reference strain *C. albicans* SC5314, generating Δlog2(MIC) values (Turnidge and Paterson 2007) for each strain–drug combination. Positive values indicate an increase in MIC relative to the reference strain, while negative values indicate enhanced susceptibility. The heatmap was constructed using Euclidean distance and Ward's hierarchical clustering (Murtagh and Legendre 2014) to group strains and antifungal agents according to similarity in their Δlog2(MIC) profiles using the R package ComplexHeatmap v.2.14.0 (Gu et al. 2016).

Biofilm formation and visualization were assessed using a microtiter plate assay and scanning electron microscopy (SEM). For biofilm quantification, isolates were streaked onto modified Sabouraud agar (2% glucose, 1% peptone, 0.5% malt extract, 0.5% yeast extract; pH 5.6) and incubated overnight at 37°C. Yeast suspensions were prepared and adjusted to 1 × 10⁷ cells mL⁻¹ in RPMI 1640 (Sigma‐Aldrich) buffered with 0.165 mol L⁻¹ MOPS [(3‐(N‐morpholino) propane sulfonic acid)]. The microtiter plate biofilm assay followed the method of Seneviratne et al. (2008) with minor modifications. Briefly, 100 µL of the suspension were dispensed into 96‐well flat‐bottom microplates and incubated at 37°C for 90 min at 75 rpm to allow cell adhesion. The supernatant was removed, wells were washed twice with PBS, fresh RPMI was added, and plates were incubated for 48 h at 37°C with medium replacement every 24 h. Total biofilm biomass was quantified by crystal violet staining according to Pierce et al. (2009), which stains viable and non‐viable cells as well as extracellular matrix. Absorbance was measured at OD₅₈₀–OD₅₉₀ (exact wavelength specified in Results). Each isolate was tested in technical triplicate across three independent experiments. Medium‐only wells served as negative controls, and *C. albicans* SC5314 was included as a susceptible reference strain.

For SEM visualization, *C. albicans* SC5314 and the strain UFMG‐CM‐Y6065 of the novel species were first grown on YPD plates for 24 h at 30°C. Cell suspensions were adjusted to 1 × 10⁷ cells mL⁻¹ in RPMI and 1 mL was added to 24‐well plates containing sterile coverslips. After 1 h of incubation at 37°C and 110 rpm to allow adhesion, wells were washed with PBS to remove non‐adherent cells, refilled with 3 mL of RPMI, and incubated for 48 h at 37°C with gentle agitation (110 rpm). Biofilms were fixed in modified Karnovsky fixative (2% paraformaldehyde, 2.5% glutaraldehyde in 0.1 M phosphate buffer) for 1 h, rinsed with phosphate buffer, stored at 4°C, and transported to the Center of Microscopy at the Universidade Federal de Minas Gerais, Belo Horizonte, MG, Brazil (http://www.microscopia.ufmg.br) for analysis using a FEI Quanta 200 FEG scanning electron microscope. Sample preparation and imaging followed the protocol described by Rai et al. (2019), with the adaptations described above. Images were corrected uniformly for contrast and brightness using the Photos application provided with Windows 11.

To assess ATP‐binding cassette (ABC)‐type drug transporter activity, glucose‐induced rhodamine 6 G efflux was measured as previously described (Maesaki et al. 1999; Gbelska et al. 2017). Isolate UFMG‐CM‐Y6066, which exhibited the highest MIC for fluconazole, was selected for this assay along with *C. albicans* SC5314. Cells were grown to logarithmic phase in YPD medium at 35°C, collected by centrifugation, and transferred to fresh YPD for 2 h of incubation at 35°C. After washing with phosphate‐buffered saline (PBS), pellets were resuspended in 10 mL of PBS with 20 μM rhodamine 6 G (glucose‐free) and incubated for 90 min to allow dye uptake. Cells were then washed again and resuspended in 750 μL of PBS. Efflux was initiated by adding 250 μL of PBS containing 8 mmol L^‐1^ glucose, while control samples received PBS without glucose. Aliquots were collected after 5, 15, and 25 min of incubation at 35°C, and fluorescence was measured in 200 μL of supernatant (excitation 527 nm, emission 555 nm; Varioskan). Additional assays were performed in the presence of fluconazole (8 μg mL^‐1^).

Adhesion to human epithelial cells was evaluated using the buccal cell adhesion (BCA) assay. Yeast isolates were cultured on YM agar for 24 h at 37°C. A yeast suspension was then prepared by adding 1 mL of culture to 4 mL of modified Sabouraud broth and incubating at 37°C for 1 h. Human oral epithelial cells were collected from healthy volunteers using sterile swabs and resuspended in 10 mL of sterile saline solution (0.85% NaCl). Cell density was adjusted to 10⁵ cells mL^‐1^ using a Neubauer chamber. For the adhesion assay, 0.5 mL of epithelial cell suspension and 0.5 mL of yeast suspension were gently mixed and incubated for 1 h at 37°C. The mixture was filtered through a 0.45 μm polycarbonate membrane and washed with ~50 mL of saline to remove non‐adherent cells. Filters were pressed onto glass slides for 10 s, air‐dried, and stained with crystal violet for 30 s. The number of yeast cells adhering to 50 epithelial cells was counted under optical microscopy, excluding overlapped or folded host cells (D'Eça Júnior et al. 2011).

## 3 Results and Discussion

### 3.1 Species Delineation and Phylogeny

**Figure 1:**
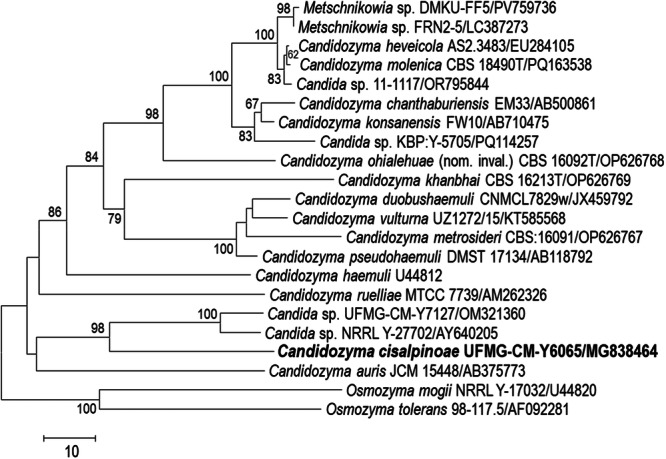
Phylogram showing the placement of *Candidozyma cisalpinoae* sp. nov. The neighbor‐joining tree was reconstructed from an alignment of 540 aligned positions of the large subunit rRNA gene D1/D2 region, using the number of substitutions as the distance metric, so as to show graphically the amount of divergence between OTUs (Operational Taxonomic Units). *Osmozyma* species are used as outgroup. Bootstraps values of 50% or more, determined from 1000 pseudoreplications, are shown.

**Figure 2:**
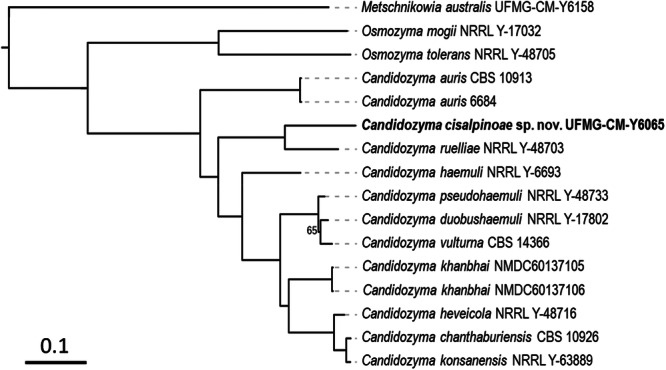
Phylogenetic tree reconstructed from the alignment of 2226 orthologous proteins of *Candidozyma cisalpinoae* compared with 12 other related species with whole genomes available of the order Serinales, with *Metschnikowia australis* UFMG‐CM‐Y6158, as the outgroup. Bootstrap values (1000 replicates) are shown only where values were less than 100%.

Sequence analysis of the D1/D2 domains placed the new species in a subclade with *Ca. auris* and two undescribed *Candidozyma* species (Figure 1). The new species differs from the holotype of *Ca. auris* in D1/D2 sequences by 30 nucleotide substitutions and 10 indels (369 bp compared), and by 46 nucleotide substitutions and 22 indels (475 bp compared) from the holotype of *Ca. ruelliae*, which was identified as a sister species by phylogenomics (Figure 2). The novel species differs from *Candidozyma* sp. UFMG‐CM‐Y7127 by 45 nucleotide substitutions and nine indels (463 bp compared), and by 48 nucleotide substitutions and eight indels from *Candidozyma* sp. NRRL Y‐27702 (466 bp compared). The ITS region distinguishes the novel species from *Ca. ruelliae* by 24 nucleotide substitutions and eight indels, and 38 nucleotide substitutions and 27 indels from *Ca. auris*. The six isolates of the novel species had identical D1/D2 and ITS sequences.

A phylogenomic analysis based on 2116 single‐copy orthologs from *Candidozyma* species for which complete genomes are available placed the new species, represented by strain UFMG‐CM‐Y6065, as a sister species to *Ca. ruelliae* (Figure 2), with *Ca. auris* occupying a more basal position relative to both species. This topology differs from that inferred from D1/D2 sequences (Figure 1), particularly with respect to the relative placement of the new species, *Ca. auris* and *Ca. ruelliae*. Differences between the two trees are expected. The analysis based on 2116 orthologs is taken to be a robust estimate of the true phylogeny, in contrast to the phylogram based on barcode sequences, whose purpose is to demonstrate the distinct nature of the new species. Comparative genomic metrics further support the placement proposed in Figure 2. The ANI value between the novel species and *Ca. ruelliae* was 77.6%, which is higher than the 73%–74% values obtained for other *Candidozoma* species (Supporting Information S1: Figure 1). Importantly, an ANI value of 77.6% add support for their separation as distinct species. The value is well below values previously reported for pairs of closely related species in some yeasts and fungi, while remaining within the range for congeneric taxa (Lachance et al. 2020; Cortimiglia et al. 2024; García‐Acero et al. 2024). This said, ANI values should be interpreted in a comparative and taxon‐specific context rather than as fixed thresholds (Riesco and Trujillo 2024).

AAI and POCP results exhibited the same pattern. AAI values between the novel species and *Ca. ruelliae* reached 81.5%, whereas values relative to the remaining species ranged from 73% to 75% (Supporting Information S1: Table 3), again consistent with interspecific, but not intergeneric, variation. Likewise, POCP values above 50% between the novel species and *Ca. ruelliae* (55.7%), and slightly lower values with the other species (54%–55.5%) (Supporting Information S1: Table 4), corroborate their shared generic placement, since POCP values below 50% are typically indicative of separation at the genus level (Liu et al. 2024). Overall, these genome‐scale metrics agree with the taxonomic framework proposed by Liu et al. (2024) for *Candidozyma* and provide strong quantitative support for recognizing the Brazilian isolates as a distinct species. Based on the combined evidence from phylogenomics, sequence divergence, and comparative genomics, we propose the name *Candidozyma cisalpinoae* sp. nov. for the new species.

**Figure 3:**
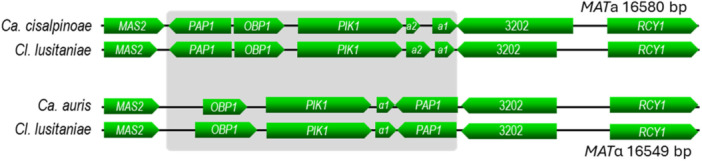
The mating locus of *Candidozyma cisalpinoae* is typical of the Metschnkowiaceae. The strain whose genome was sequenced has mating type a, which is collinear with that of *Clavispora lusitaniae*. An alignment of the α locus of *Ca. auris* is shown for comparison. The genes are drawn to scale, although the presence of indels may cause distortions in the sizes of individual genes. The loci for other *Candidozyma* species show no significant differences in collinearity.

Although *Ca. cisalpinoae* did not mate or form asci when mixed in pairs on various sporulation media, examination of the genome of strain UFMG‐CM‐Y6065 showed the presence of a complete mating locus containing the non‐sex genes *PIC1*, *OBP1* and *PAP1*, and flanked by genes *MAS2* and *RCY1*, as is typical of Metschnikowiaceae (Figure 3) except for *Australozyma* species (Lachance et al. 2025). The strain has mating type **a**. As is also typical, the *MF* **a** gene was present in nine dispersed copies differing by 5–18 substitutions and translating into five nascent peptides, although the processed pheromones were identical, with the sequence NLNKAAGRGAVPFTCTIV. In other species of the genus, the number of genes varied from one to 15, with each species generating one or two versions of the **a**‐pheromone. The α‑pheromone was equally typical of the family with the presence of a single open reading frame encoding four tandem copies differing by 11–15 substitutions, but all translating into the same processed peptide, with the sequence WGWLRFWPGOPFV. Other species had one or two copies of the gene, and the number of coding sequences in each gene varied from three to five. *Candidozyma cisalpinoae* shared the **a** mating type with *Ca. vulturna*, *Ca. auris* and *Ca. pseudohaemuli*. The last two species also feature individuals of mating type α, which is the only mating type so far identified in the remaining six species for which genome sequences are available. Large numbers of genome sequences have been determined only for *Ca. auris*. In that species, Muñoz et al. (2018) observed a 37:14 distribution of α and **a** mating types, which show a strong association with four phylogenetic subgroups and geography, indicating that proliferation of clinical specimens is overwhelmingly asexual. Wang and Xu (2022) looked for evidence of recombination by analysis of 1285 strains and concluded that sexual reproduction probably occurred more frequently before the split of the species into its five currently known clades, but that recombination in the current species cannot be ruled out. The paucity of genome data for other *Candidozyma* species limits our ability to speculate on the distribution of mating types in the genus as a whole. This notwithstanding, the presence of mating type **a** in at least four of the 10 species, and importantly, the integrity of mating loci and pheromone genes, which we verified here, nearly guarantees that a sexual cycle will eventually be discovered in *Candidozyma* species, including *Ca. cisalpinoae*. This will require intensified sampling and might benefit from the development of a PCR‐based test to determine mating types, which would ease the selection of strains that have the potential to mate and form asci. Hitherto, sexual cycles of Metschnikowiaceae have been demonstrated in many *Metschnikowia* species, two *Clavispora* species, and the single *Danielia* species, but remain to be documented in the many newly circumscribed genera of the family (Liu et al. 2024), most of which appear to possess the required genetic equipment.

The novel species can be differentiated from *Ca. ruelliae*, *Ca. haemuli* and *Ca. auris* by the assimilation of D‐ribose and ribitol, which are negative for *Ca. cisalpinoae* and positive for those species. Furthermore, *Ca. cisalpinoae* can also be differentiated from other phylogenetically related species by weak growth on citrate and fermentation of maltose (Supporting Information S1: Table 5).

### 3.2 Ecological Origins and Environmental Sources of *Candidozyma* Species

*Candidozyma* species occupy an intriguing ecological niche, as the genus includes both environmental species associated with plant substrates and clinically relevant opportunistic pathogens (Zandijk et al. 2025). Environmental representatives such as *Ca. chanthaburiensis, Ca. ruelliae*, *Ca. heveicola*, *Ca*. *metrosideri* and *Ca. ohialehuae* have been recovered from nectar‐rich flowering plant species, decaying wood and tree barks (Limtong and Yongmanitchai 2010; Klaps et al. 2020; Liu et al. 2024). Floral microhabitats such as nectar and floral surfaces represent chemically and physically heterogeneous environments. Nectar properties vary widely among plant species and are strongly influenced by pollination syndrome, including nectar volume, sugar concentration, and sugar composition. For instance, ornithophile flowers typically produce more dilute, hexose‐rich nectars, whereas insect‐pollinated flowers often exhibit higher sugar concentrations and sucrose‐dominant nectars (Mittelbach et al. 2015). These differences have important consequences for yeast inoculation, colonization, and persistence in floral habitats. Despite this heterogeneity, many floral microhabitats impose intermittent osmotic, chemical, and thermal stresses, particularly in flowers producing concentrated nectars or exposed to fluctuating environmental conditions. These conditions may select yeasts capable of rapid growth, stress tolerance, and competitive interactions which are traits that overlap with phenotypes associated with opportunistic pathogenicity in humans (Herrera et al. 2010; Lachance 2016; Rosa et al. 2023; Cosio et al. 2025). We did not determine the nectar properties of the studied *Ipomoea* flowers. Galetto and Bernardello (2004) studied the floral nectaries and nectar features of six *Ipomoea* species in Argentina with differences in their pollinator guilds. These authors showed that all nectar samples contained amino acids and sugars. Most species had sucrose‐dominant nectars, a sugar assimilated by *Ca. cisalpinoae*. This novel yeast species was isolated from an unidentified species of *Ipomoea*, and we did not know the chemical composition of the flowers of this species. *Ipomoea* flowers are visited mostly by drosophilids, bees and nitidulid beetles, and these insects are responsible for vectoring the yeasts among different flowers. The association of *Candidozyma* species with pollinating insects and *Ipomoea* flowers further suggests dispersal through floral networks and insect‐mediated transmission (Brysch‐Herzberg 2004; Lachance et al. 2001).

Some authors have proposed that the repeated emergence of pathogenicity within this clade may reflect preadaptation to thermal, osmotic and chemical stress common in plant or insect‐associated microhabitats (Casadevall et al. 2019; Borman and Johnson 2020). Indeed, several environmental species, including *Ca. ruelliae* and *Ca. ohialehuae*, exhibit high thermotolerance and resistance to antifungal compounds naturally produced by plants or microbial competitors (Klaps et al. 2020; Liu et al. 2024). Given that *Ca. auris* is hypothesized to have existed as a plant saprotroph in specialized ecosystems, such as wetlands, that adapted to higher temperatures prior to its emergence in clinical settings (Jackson et al. 2019; Casadevall et al. 2021), the discovery of *Ca. cisalpinoae* in flowers reinforces the relevance of floral microhabitats as reservoirs of yeasts possessing traits that, under appropriate ecological or evolutionary pressures, may facilitate opportunistic pathogenicity. Thus, the ecological context of *Candidozyma*, bridging plant‐associated environments and clinically important lineages, underscores the importance of continued environmental sampling to trace the origins, genetic diversity, and adaptive pathways that shape the evolution of virulence within this group. The isolates of this yeast were obtained in a region with daytime temperatures above 30°C for most of the year. These temperatures may be acting as additional ecological factor to select for thermotolerant yeasts, such as the species described in this work.

### 3.3 *Ca. cisalpinoae* Shows Multidrug Resistance and Virulence‐Associated Phenotypes

**Figure 4:**
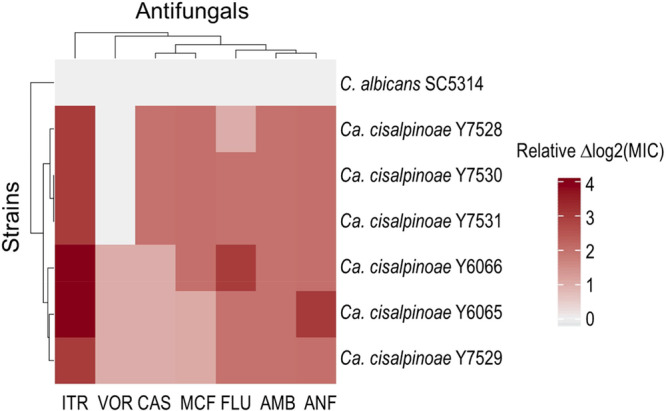
Heatmap with relative antifungal susceptibility profiles expressed as log2(MIC) values for seven antifungal agents: caspofungin (CAS), micafungin (MCF), amphotericin B (AMB), itraconazole (ITR), anidulafungin (ANF), fluconazole (FLU), and voriconazole (VOR). Red tones correspond to higher MICs (reduced susceptibility) relative to the control strain *C. albicans* SC5314, while blue tones indicate lower MICs (higher susceptibility). The results reveal a consistent pattern of reduced susceptibility to azoles and amphotericin B in *Candidozyma cisalpinoae*, while susceptibility to echinocandins (CAS, MCF and ANF) is preserved across the strains.

The in vitro antifungal susceptibility of six isolates was tested for amphotericin B, fluconazole, itraconazole, voriconazole, anidulafungin, caspofungin and micafungin. The heatmap (Figure 4) shows the relative change in antifungal susceptibility of *Ca. cisalpinoae* strains compared to *C. albicans* SC5314, expressed as Δlog2(MIC). The consistent positive Δlog2 values (red colors) observed across most azoles and amphotericin B indicate that all *Ca. cisalpinoae* isolates require higher drug concentrations for growth inhibition than *C. albicans* tested, revealing a pattern of reduced susceptibility. Itraconazole displays the largest increases in Δlog2(MIC) among all drugs, followed by fluconazole, reflecting the stronger relative resistance of these strains to triazoles. Interestingly, voriconazole, a second generation triazole, exhibited elevated MIC values for most *Ca. cisalpinoae* isolates. Considering the clinical breakpoints established for *C. albicans*, these values would fall within the dose‐dependent susceptibility range (CLSI 2008). In contrast, Δlog2(MIC) values for caspofungin, micafungin and anidulafungin remain close to the control strain (light gray = 0), indicating uniformly high susceptibility to echinocandins across isolates (Supporting Information S1: Table 6). The hierarchical clustering groups the three echinocandins together as the most active compounds and splits them from azoles and amphotericin B, which cluster according to their reduced efficacy. These results show that *Ca. cisalpinoae* exhibits intrinsic reduced susceptibility to azoles and amphotericin B, while being susceptible to echinocandins. However, there are no clinical breakpoints or epidemiological cutoff values available for *Ca. cisalpinoae*. Following the CLSI criteria applicable to *C. albicans* and as per CDC *Ca. auris* tentative MIC breakpoints, the results for *Ca. cisalpinoae* suggest resistance to fluconazole, itraconazole and amphotericin B and dose‐dependent for voriconazole (CLSI 2008; CDC 2024). Interestingly, despite being isolated from an environmental source, *Ca. cisalpinoae* shows a resistance profile comparable to other clinically relevant species of the genus, such as *Ca. haemuli* or *Ca. auris* (Silva et al. 2020; Rybak et al. 2022). Combined with the ability to grow at 37°C, this profile suggests that this yeast may have opportunistic pathogenic potential for animals, including humans.

**Figure 5:**
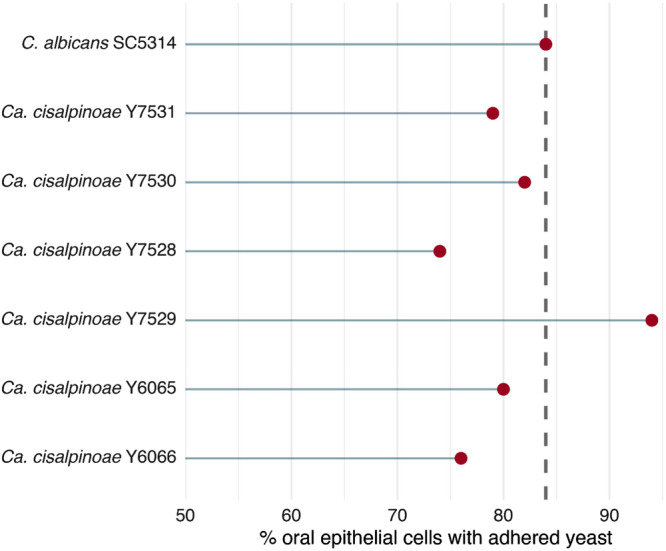
Percentage of epithelial cells with adhered yeast in buccal cell adhesion assays. Values represent the proportion of oral epithelial cells that showed at least one attached yeast after interaction with each isolate. *Candida albicans* SC5314 was included as a control. Some *Candidozyma cisalpinoae* isolates displayed adhesion levels similar to or exceeding those of the reference strain SC5314.

The ability of yeasts to adhere to the oral mucosa may be linked to their pathogenicity, as it is a critical step in the process of infection by *Candida* species and other opportunistic yeasts, being essential for both colonization and subsequent induction of disease (Talapko et al. 2021). Adherence to epithelial surfaces is also considered an important ecological trait, as it allows yeasts to persist in host‐associated niches, resist mechanical removal, and compete with resident microbiota, and in pathogenic species this step frequently precedes tissue invasion, biofilm development, and immune evasion (Lass‐Flörl et al. 2024). This process occurs due to the expression of cell‐surface adhesion proteins, such as adhesins. In this work, the isolates of *Ca. cisalpinoae* showed 74%–94% adhesion to oral epithelial cells (Figure 5, Supporting Information S1: Table 7), showing that this yeast presents the ability to adhere to these cells. The isolate *C. albicans* SC5314 was used as a control in the experiments on buccal cell adhesion, as this species is widely studied as to its cell adhesion capacity, with a percentage of adhered yeast of 84%. Several studies show that *C. albicans* has more developed and efficient adhesion mechanisms compared to other opportunistic *Candida* species, which facilitates its colonization of different surfaces, including medical materials and host tissues (Sionov et al. 2020; Martin et al. 2021).

**Figure 6:**
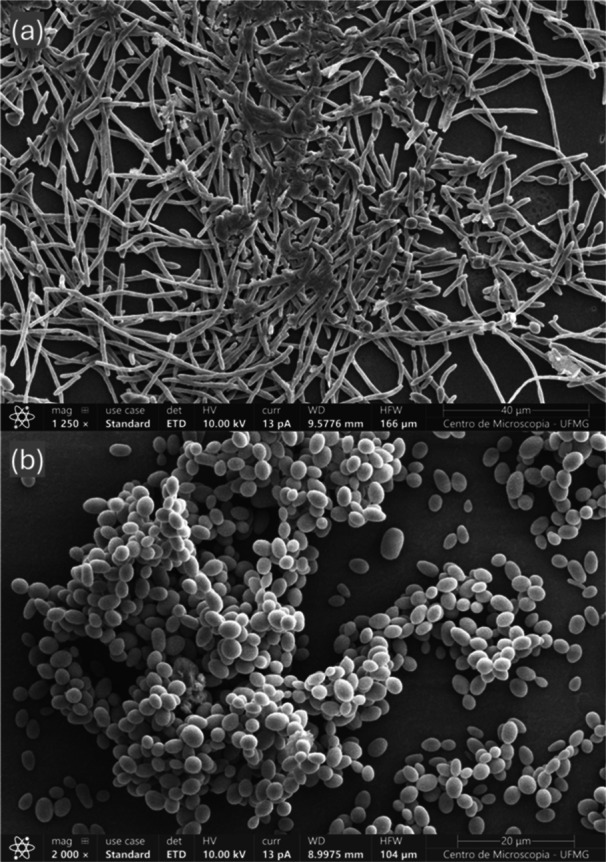
Scanning Electron Microscopy of biofilms formed by *Candida albicans* SC5314 (a) and *Candidozyma cisalpinoae* UFMG‐CM‐Y6065 (b) after 48 h at 37°C. *C. albicans* shows extensive filamentation, whereas *Ca. cisalpinoae* forms dense clusters of blastoconidia without hyphae or pseudohyphae.

Among the properties associated with the pathogenicity of a microorganism are the ability to form biofilms and efflux pumps (Egue et al. 2018; Nett and Andes 2020). The ability to form biofilms is an important characteristic for the survival and pathogenicity of yeasts, especially in hostile environments. It is important to note that strains with a greater capacity for biofilm production may represent clinical challenges, as biofilms enhance tolerance to antifungal agents and host immune responses (Malinovská et al. 2023). In *C. albicans*, for instance, non‐sex genes located within the mating‐type locus have been shown to participate in a/α biofilm formation, including the development of an impermeable matrix and increased fluconazole resistance, underscoring the contribution of biofilm architecture to pathogenicity (Srikantha et al. 2012). The same isolates tested for adhesion were also evaluated for biofilm formation (Supporting Information S1: Table 8). All strains formed biofilms after 48 h at 37°C, with crystal violet absorbance reflecting total biomass. The reference strain *C. albicans* SC5314 showed a mean absorbance of 0.234. All *Candidozyma* isolates produced comparable or slightly higher biomass, ranging from 0.247 to 0.397. Although some strains (UFMG‐CM‐Y7530 and UFMG‐CM‐Y7529) showed higher values, statistical analysis indicated no significant differences relative to *C. albicans* SC5314 (*p* > 0.05). To complement the microplate assay, biofilms formed by UFMG‐CM‐Y6065 and *C. albicans* SC5314 were examined by scanning electron microscopy. SEM imaging showed that *Candidozyma* UFMG‐CM‐Y6065 produced a compact three‐dimensional biofilm composed of densely aggregated blastoconidia, without observable hyphae or pseudohyphae (Figure 6). In contrast, *C. albicans* SC5314 formed a biofilm dominated by extensive hyphal and pseudohyphal structures. Under the SEM preparation conditions used, extracellular matrix material could not be clearly visualized in either species.

**Figure 7:**
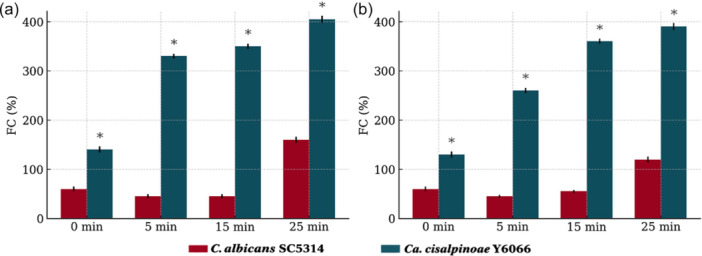
Rhodamine 6G (R6G) efflux over time in *Candidozyma cisalpinoae* UFMG‐CM‐Y6066 and *Candida albicans* SC5314 strains, with data presented as percentage of fluorescent cells (%FC) after the addition of 8 mM glucose. a; Rhodamine 6G (R6G) efflux over time in *Ca. cisalpinoae* UFMG‐CM‐Y6066 and *C. albicans* SC5314 strains, with data presented as percentage of fluorescent cells (%FC) after the addition of 8 mM glucose. b: Rhodamine 6G (R6G) efflux over time in UFMG‐CM‐Y6066 and SC5314 cells treated with FLC, also expressed as percentage of fluorescent cells (%FC) after the addition of 8 mM glucose. Asterisks denote statistically significant differences between UFMG‐CM‐Y6066 and SC5314 cells.

To investigate whether azole resistance in this novel species could be associated with efflux‐mediated drug tolerance, as previously observed in members of the *Ca. auris–Ca. haemuli* clade (Silva et al. 2020), we evaluated ABC‐type transporter activity using the rhodamine 6 G (R6G) efflux assay. R6G is a fluorescent substrate commonly extruded by ABC family transporters, the same class of drug pumps associated with azole efflux in yeasts. Isolate UFMG‐CM‐Y6066, which presented the highest MIC value for fluconazole, showed strong glucose‐induced R6G efflux, consistent with active ABC‐type transport. Efflux levels in *Ca. cisalpinoae* UFMG‐CM‐Y6066 were significantly higher than those observed for *C. albicans* SC5314, with 5.54‐, 7.57‐, and 2.38‐fold increases at 5, 15, and 25 min, respectively (*p* < 0.05; Figure 6a). These results suggest that active efflux contributes to the reduced susceptibility of *Ca. cisalpinoae* to azoles. Interestingly, fluconazole treatment did not alter glucose‐induced R6G efflux in UFMG‐CM‐Y6066 (Figure 6b), indicating that pump activity was not upregulated by the drug under the experimental conditions tested. A similar phenotype was reported for *Ca. haemuli* (Silva et al. 2020), suggesting that constitutive efflux activity may be a conserved trait within this lineage. Together, these findings support the hypothesis that ABC‐type transporters play an important role in azole tolerance in *Ca. cisalpinoae*, potentially contributing to its intrinsic reduced susceptibility (Figure 7).

## 4 Conclusions and Taxonomy

Together, our results demonstrate that *Ca. cisalpinoae* represents a distinct species within the *Candidozyma* clade, supported by multilocus phylogeny, genome‐scale metrics, and phenotypic traits. Although isolated from floral substrates, *Ca. cisalpinoae* displays several traits commonly associated with opportunistic pathogenic yeasts, including thermotolerance, adhesion to epithelial cells, biofilm formation, reduced susceptibility to azoles and amphotericin B, and active ABC‐type efflux of rhodamine 6G. The combination of intrinsic drug tolerance and the ability to grow at 37°C suggests that *Ca. cisalpinoae* may possess latent pathogenic potential, similar to other environmental members of this group that have emerged as opportunistic pathogens in humans and animals. The discovery of this species in flowers reinforces the role of plant‐associated niches as reservoirs of yeasts that harbor physiological and genetic traits predisposing them to host adaptation. As global reports of environmental opportunistic yeasts continue to rise, the characterization of *Ca. cisalpinoae* expands our understanding of diversity, ecology, and the evolutionary paths that may lead to pathogenicity within *Candidozyma*. Continued environmental monitoring and comparative genomic analyses will be essential to elucidate the mechanisms by which environmental lineages acquire virulence traits and to assess their clinical relevance.

Description of *Candidozyma cisalpinoae* sp. nov. A. P. O. Tironi, K. O. Barros, L. F. A. Santana, D. L. Souza, A. R. O. Santos, G. R. Ávila, T. M. Batista, G. R. Franco, S. Johann, Lachance & Rosa sp. nov.

*Candidozyma cisalpinoae* (ci.sal.pi´no.ae. N. L. gen. n. *cisalpinoae* of Cisalpino, in honor of Prof. Patrícia Silva Cisalpino, in recognition of her contributions to the study of pathogenic fungi in Brazil).

**Figure 8:**
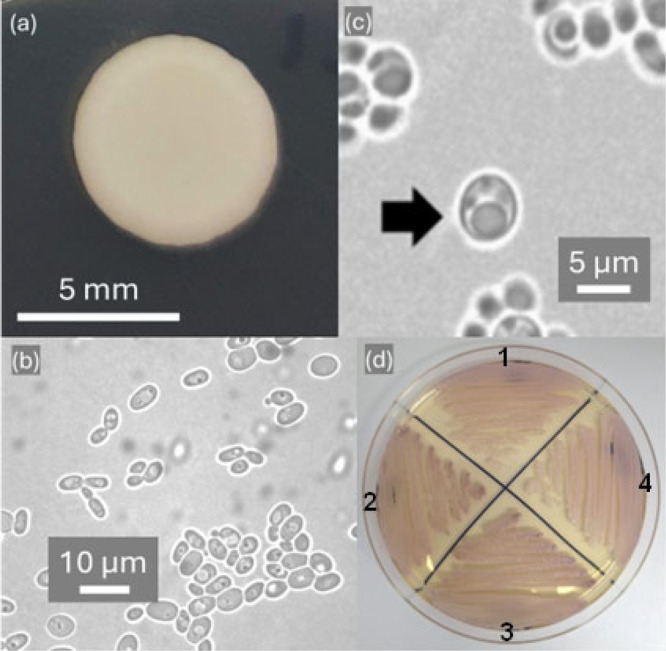
Morphology of *Candidozyma cisalpinoae* UFMG‐CM‐Y6065. (a) Colony morphology on YM agar after incubation for 3 days at 25°C. (b) Yeast cells after 3 days on YM agar. (c) Yeast cells from a 7‐day old culture on acetate agar incubated at 25°C showing lipid droplets of larger cell (arrow). (d) Colonies grown on CHROMagar Candida™ after 3 days at 37°C: (1) *Ca. haemuli* UFMG‐CM‐Y7534 (2) *Ca. auris* COL01B (3) *Ca. cisalpinoae* UFMG‐CM‐Y6066, (4) *Ca. cisalpinoae* UFMG‐CM‐Y6065.

Cells are ovoid to ellipsoidal, (2–3.3 × 3–5 μm) and reproduce by multilateral budding on YM agar after incubation for 3 days at 25°C (Figure 8). After 72 h of incubation at 25°C on YM agar, colonies appear whitish, smooth, shiny, soft, and exhibit an entire margin (Figure 8). Pseudohyphae and true hyphae are not formed on Spider agar or cornmeal agar after 15 days of incubation. Lipid droplets are present after 7 days on acetate agar at 25°C (Figure 8c). On CHROMagar *Candida*™, colonies are light cream to lilac (Figure 8d). In yeast extract (0.5%) ‐ glucose broth (2%), sediment forms after 1 month, with a pellicle and a ring. Budding is multilateral. Mating or ascus formation are not observed in mixed cultures on cornmeal, GY, acetate, YM, or malt extract agar after 12 weeks of incubation at 18°C or 25°C. Genome analysis shows that the species is haplontic and heterothallic. Glucose fermentation is positive, with delayed fermentation of maltose. Assimilation of carbon compounds: Positive for glucose, sucrose, galactose, trehalose, maltose, melizitose, cellobiose, lactose (weak), L‐sorbose (weak), L‐rhamnose (weak), L‐arabinose (weak), ethanol (weak), glycerol, D‐mannitol, D‐glucitol, citrate (weak) and xylitol (weak). Inulin, raffinose, melibiose, soluble starch, salicin, D‐xylose, D‐arabinose, D‐ribose, methanol, erythritol, ribitol, myo‐inositol, DL‐lactate, succinate, D‐gluconate, D‐glucosamine, hexadecane, ethyl acetate, isopropanol and acetone are not assimilated. Assimilation of nitrogen compounds: positive for lysine and negative for nitrate and nitrite. Growth in amino‐acid‐free medium is positive. Growth at 37°C is positive. The maximum temperature for growth is 42°C. Growth on YM agar with 10% sodium chloride and 50% glucose/yeast extract (0.5%) is negative. Acid production is positive. Starch‐like compounds are not produced. In cycloheximide 100 µg mL^‐1^ growth is positive weak. Urease activity is negative. Diazonium Blue B reaction is negative. The habitat is flowers of *Ipomoea* sp. collected in the Cerrado ecosystem of state Tocantins, Brazil. The holotype of *Candidozyma cisalpinoae* sp. nov. is CBS 16108, which is preserved in a metabolically inactive state in the CBS Yeast Collection of the Westerdijk Fungal Biodiversity Institute. It was isolated from flower of *Ipomoea* sp. collected in the Cerrado ecosystem of state Tocantins, Brazil. An isotype of *Candidozyma cisalpinoae* sp. nov., UFMG‐CM‐Y6065, is deposited in the Collection of Microorganisms and Cells of Federal University of Minas Gerais (Coleção de Microrganismos e Células da Universidade Federal de Minas Gerais, UFMG), Belo Horizonte, Minas Gerais, Brazil. Parathypes of *Candidozyma cisalpinoae* are UFMG‐CM‐Y6066, UFMG‐CM‐Y7528, UFMG‐CM‐Y7529, UFMG‐CM‐Y7530 and. UFMG‐CM‐Y7531. The MycoBank number is 861366. The GenBank/EMBL/DDBJ accession numbers for the D1/D2 domain of the large subunitrRNA gene sequence, ITS‐5.8S region, of the small‐subunit rRNA gene sequence of strain UFMG‐CM‐Y6065 are MG838464 and PP889739, respectively. The Whole Genome Shotgun project of strain *C. cisalpinoae* has been deposited at GenBank under accession PRJNA1180885.

## Author Contributions

Anna Paula O. Tironi, Katharina O. Barros, Carlos A. Rosa and Susana Johann conceived and designed the study. Carlos A. Rosa collected the samples and isolated the yeasts. Anna Paula O. Tironi, Luiz Felipe A. Santana, Giovana R. Ávila, Daniela L. Souza and Ana Raquel O. Santos performed experiments. Anna Paula O. Tironi, Katharina O. Barros, Carlos A. Rosa, Thiago M. Batista, and Marc‐André Lachance analyzed data. Anna Paula O. Tironi and Katharina O. Barros wrote the paper. Anna Paula O. Tironi, Katharina O. Barros, Thiago M. Batista, Marc‐André Lachance, Glória R. Franco, Carlos A. Rosa and Susana Johann edited the manuscript, and all co‐authors approved it.

## Conflicts of Interest

The authors declare no conflicts of interest.

## Supporting information


**Figure S1:** Heatmap generated with OrthoANI values calculated from the OAT software. **Table S1:** Publicly available genomes used in the phylogenomic analysis. **Table S2:** Genome properties for *Candidozyma cisalpinoae.* **Table S3:** AAI (Average Amino Acid Identity) values between *Candidozyma cisalpinoae* and other related *Candidozyma* and *Osmozyma* yeast species. **Table S4:** Percentage of Conserved Proteins (POCP) among different *Candidozyma* species and other related species. **Table S5:** Differential physiological characteristics among *Candidozyma cisalpinoae* sp. *nov*, and the other phylogenetically related *Candidozyma* species. **Table S6:** MIC (Microdilution Inhibitory Concentration) for *Candida albicans* and each isolate of *Candidozyma cisalpinoae*. **Table S7:** Adhesion of yeasts to oral epithelial cells for *Candida albicans* and each isolate of *Candidozyma cisalpinoae*. **Table S8:** Analysis of Biofilm formation (crystal violet absorbance) for *Candida albicans* and each isolate of *Candidozyma cisalpinoae*.

## Data Availability

The data that support the findings of this study are available from the corresponding author upon reasonable request.
