# Supplementary material for: Candidozyma cisalpinoae sp. nov., a Genomically Distinct, Flower‐Associated Yeast, Resistant to Azoles and Exhibiting Pathogenicity‐Related Traits
Source: Yeast. 2026 Mar 5;43(1-2):25–37. doi: 10.1002/yea.70012 (PMC13067812; doi:10.1002/yea.70012)
Supplement: Supplementary file 1 — Figure S1: Heatmap generated with OrthoANI values calculated from the OAT software. Table S1: Publicly available genomes used in the phylogenomic analysis. Table S2: Genome properties for Candidozyma cisalpinoae. Table S3: AAI (Average Amino Acid Identity) values between Candidozyma cisalpinoae and other related Candidozyma and Osmozyma yeast species. Table S4: Percentage of Conserved Proteins (POCP) among different Candidozyma species and other related species. Table S5: Differential physiological characteristics among Candidozyma cisalpinoae sp. nov, and the other phylogenetically related Candidozyma species. Table S6: MIC (Microdilution Inhibitory Concentration) for Candida albicans and each isolate of Candidozyma cisalpinoae. Table S7: Adhesion of yeasts to oral epithelial cells for Candida albicans and each isolate of Candidozyma cisalpinoae. Table S8: Analysis of Biofilm formation (crystal violet absorbance) for Candida albicans and each isolate of Candidozyma cisalpinoae. [file YEA-43-25-s001.docx]

***Candidozyma cisalpinoae* sp. nov., a genomically distinct flower-associated yeast resistant to azoles and exhibiting pathogenicity-related traits**

Anna Paula O. Tironi^1^, Katharina O. Barros^1^, Luiz Felipe A. Santana^1^, Daniela L. Souza^3^, Ana Raquel O. Santos^1^, Giovana R. Ávila^1^, Thiago M. Batista^2^, Glória R. Franco^3^, Raphael S. Pimenta^4^, Paula B. Morais^4^, Marc-André Lachance^5^, Carlos A. Rosa^1#^, Susana Johann^1#^

^1^Departamento de Microbiologia, ICB, C.P. 486, Universidade Federal de Minas Gerais, Belo Horizonte, MG, 31270-901, Brazil

^2^ Instituto Nacional da Mata Atlântica, Santa Teresa, ES, 29650-000, Brazil

^3^Departamento de Bioquímica e Imunologia, ICB, C.P. 486, Universidade Federal de Minas Gerais, Belo Horizonte, MG, 31270-901, Brazil

^4^Laboratorio de Microbiologia Ambiental e Biotecnologia, Universidade Federal do Tocantins, Palmas, TO 77020-220, Brazil

^5^Department of Biology, University of Western Ontario, London, ON N6A 5B7, Canada

**Correspondence:**

Carlos A. Rosa

[carlrosa@icb.ufmg.br](mailto:carlrosa@icb.ufmg.br)

Susana Johann

[sjohann@icb.ufmg.br](mailto:sjohann@icb.ufmg.br)

**Keywords:** *Candidozyma cisalpinoae* sp. nov.; tropical flowers; virulence factors.

Table S1: Publicly available genomes used in the phylogenomic analysis.

| **Species on the phylogenomic tree** | **GenBank accession number** |
| --- | --- |
| *Candidozyma auris* CBS 10913 | GCA_030581515.1 |
| *Candidozyma auris* B11220 | GCA_003013715.2 |
| *Candidozyma cisalpinoae* UFMG-CM-Y6065 | PRJNA1180885 |
| *Candidozyma ruelliae* NRRL Y-48703 | GCA_030582895.1 |
| *Candidozyma haemuli* NRRL Y-6693 | GCA_030569475.1 |
| *Candidozyma pseudohaemuli* NRRL Y-48733 | GCA_030573385.1 |
| *Candidozyma duobushaemuli* NRRL Y-17802 | GCA_030575055.1 |
| *Candidozyma khanbhai* CBS 16213 | NMDC60137105 |
| *Candidozyma khanbhai* CBS 16555 | NMDC60137106 |
| *Candidozyma vulturna* CBS 14366 | GCA_026585945.1 |
| *Candidozyma heveicola* NRRL Y-48716 | GCA_003708405.2 |
| *Candidozyma chanthaburiensis* CBS 10926 | GCA_030579055.1 |
| *Candidozyma konsanensis* NRRL Y-63889 | GCA_030563505.1 |
| *Osmozyma tolerans* NRRL Y-48705 | GCA_030582955.1 |
| *Osmozyma mogii* NRRL Y-17032 | GCA_030573315.1 |
| *Metschnikowia australis* UFMG-CM-Y6158 | GCA_002073855.1 |

Table S2. Genome properties for *Candidozyma cisalpinoae.*

| **Category** | **Information** |
| --- | --- |
| **Whole genome sequencing** |  |
| Paired-end reads | 4,888,558 (2 × 301) |
| Total bases | 2,938,893,579 |
| Single-end reads | 3,803,676 |
| Read lengths | 50 to 592 bases |
| Total bases (single-end reads) | 1,692,691,710 |
| Estimated coverage | 115× |
| **Genome assembly** |  |
| Total size | 14,680,035 bp |
| Number of contigs (>505 bp) | 146 |
| Largest contig | 2,600,174 bp |
| GC% | 47.90% |
| Mean contig length | 100,548 bp |
| N50 | 1,178,541 bp |
| L50 | 5 contigs |
| Completeness | 99% (saccharomycetes_odb10 = 2,137 BUSCOs) |
| **Gene prediction** |  |
| Protein-coding genes | 5,683 |
| Proteins in SwissProt database | 4,455 |
| Proteins in TrEMBL database | 980 |
| Total annotated proteins | 5,435 (95.6% of predicted proteins) |
| Proteins annotated with InterProScan | 5,416 (including signatures, functional domains, and families) |


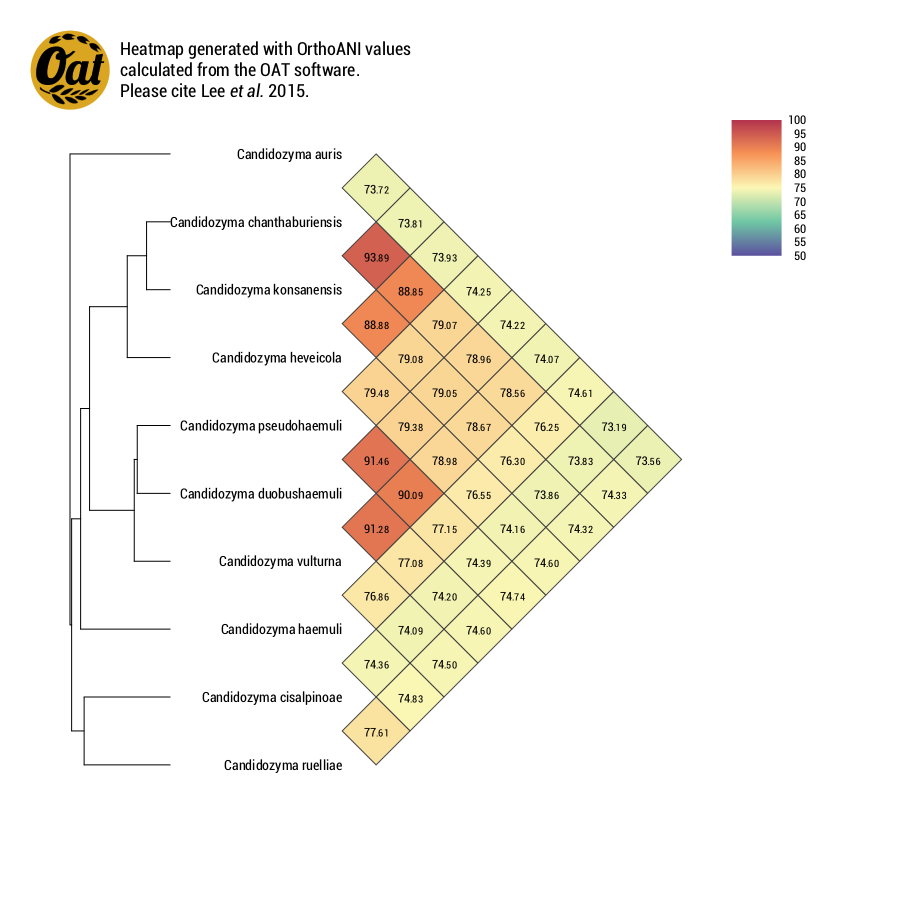


Figure S1: Heatmap generated with OrthoANI values ​​calculated from the OAT software. Each cell in the map represents the percentage of ANI (Average Nucleotide Identity) similarity between species related to *Candidozyma cisalpinoae*, identified on the sides of the cell. Only the holotype strains were used in this analysis. The colors follow the scale shown on the right, where more intense shades of red indicate greater similarity, while shades of yellow indicate lesser similarity.

Table S3: AAI (Average Amino Acid Identity) values ​​between *Candidozyma cisalpinoae* and other related *Candidozyma* and *Osmozyma* yeast species. AAI values ​​range from 81.7-64.2% for most species, with a higher similarity of 81.7% observed between *Ca. cisalpinoae* and *Ca. ruelliae*, indicating a close phylogenetic relationship.

| Genome A | Genes in A | Genome B | Genes in B | Orthologous genes | Mean AAI | Std^*^  AAI | Orthologous fraction (OF) |
| --- | --- | --- | --- | --- | --- | --- | --- |
| *Ca. haemuli*  NRRL Y-6693 | 11416 | *Ca. auris*__b11220 | 10710 | 4759 | 77.06 | 13.11 | 44.44 |
| *Os.* _*tolerans*_NRRL Y-48705 | 10869 | *Ca. haemuli*_NRRL Y-6693 | 11416 | 4102 | 65 | 15.21 | 37.74 |
| *Os.* _*tolerans*_NRRL Y-48705 | 10869 | *Ca. auris*__b11220 | 10710 | 3967 | 64.67 | 15.34 | 37.04 |
| *Ca. vulturna*_CBS 14366 | 11303 | *Os. tolerans*_NRRL Y-48705 | 10869 | 3853 | 65.4 | 15.31 | 35.45 |
| *Ca. vulturna*_CBS 14366 | 11303 | *Ca. haemuli*_NRRL Y-6693 | 11416 | 4770 | 81 | 12.41 | 42.2 |
| *Ca. vulturna*_CBS 14366 | 11303 | *Ca. auris*__b11220 | 10710 | 4462 | 76.26 | 13.53 | 41.66 |
| *Ca. duobushaemuli*_NRRL Y-17802 | 10456 | *Ca. vulturna*_CBS 14366 | 11303 | 5392 | 94.45 | 7.09 | 51.57 |
| *Ca. duobushaemuli_*NRRL Y-17802 | 10456 | *Os. tolerans*_NRRL Y-48705 | 10869 | 4021 | 65.03 | 15.16 | 38.46 |
| *Ca. duobushaemuli*_NRRL Y-17802 | 10456 | *Ca. haemuli*_NRRL Y-6693 | 11416 | 4962 | 80.77 | 12.43 | 47.46 |
| *Ca. duobushaemuli*_NRRL Y-17802 | 10456 | *Ca. auris*__b11220 | 10710 | 4641 | 76.04 | 13.41 | 44.39 |
| *Ca. pseudohaemuli*_NRRL Y-48733 | 10441 | *Ca. duobushaemuli*_NRRL Y-17802 | 10456 | 5633 | 94.71 | 7.24 | 53.95 |
| *Ca. pseudohaemuli*_NRRL Y-48733 | 10441 | *Ca. vulturna*_CBS 14366 | 11303 | 5399 | 94.13 | 7.61 | 51.71 |
| *Ca. pseudohaemuli*_NRRL Y-48733 | 10441 | *Os. tolerans*_NRRL Y-48705 | 10869 | 4000 | 64.97 | 15.22 | 38.31 |
| *Ca. pseudohaemuli*_NRRL Y-48733 | 10441 | *Ca. haemuli*_NRRL Y-6693 | 11416 | 4959 | 80.92 | 12.43 | 47.5 |
| *Ca. pseudohaemuli*_NRRL Y-48733 | 10441 | *Ca. auris*__b11220 | 10710 | 4643 | 76.14 | 13.44 | 44.47 |
| *Ca. heveicola*_NRRL Y-48716 | 10482 | *Ca. pseudohaemuli*_NRRL Y-48733 | 10441 | 4960 | 84.25 | 10.86 | 47.51 |
| *Ca. heveicola*_NRRL Y-48716 | 10482 | *Ca. duobushaemuli*_NRRL Y-17802 | 10456 | 4963 | 84.13 | 10.89 | 47.47 |
| *Ca. heveicola*_NRRL Y-48716 | 10482 | *Ca. vulturna*_CBS 14366 | 11303 | 4761 | 84.13 | 10.89 | 45.42 |
| *Ca. heveicola*_NRRL Y-48716 | 10482 | *Os. _tolerans*_NRRL Y-48705 | 10869 | 3995 | 64.71 | 15.08 | 38.11 |
| *Ca. heveicola*_NRRL Y-48716 | 10482 | *Ca. haemuli*_NRRL Y-6693 | 11416 | 4907 | 79.8 | 12.2 | 46.81 |
| *Ca. heveicola*_NRRL Y-48716 | 10482 | *Ca. auris*__b11220 | 10710 | 4613 | 75.42 | 13.24 | 44.01 |
| *Ca. ruelliae*_NRRL Y-48703 | 11134 | *Ca. heveicola*_NRRL Y-48716 | 10482 | 4680 | 75.2 | 13.13 | 44.65 |
| *Ca. ruelliae*_NRRL Y-48703 | 11134 | *Ca. pseudohaemuli*_NRRL Y-48733 | 10441 | 4700 | 75.89 | 13.2 | 45.01 |
| *Ca. ruelliae*_NRRL Y-48703 | 11134 | *Ca. duobushaemuli*_NRRL Y-17802 | 10456 | 4707 | 75.82 | 13.12 | 45.02 |
| *Ca. ruelliae*_NRRL Y-48703 | 11134 | *Ca. vulturna*_CBS 14366 | 11303 | 4513 | 76.12 | 13.15 | 40.53 |
| *Ca. ruelliae*_NRRL Y-48703 | 11134 | *Os. tolerans*_NRRL Y-48705 | 10869 | 3969 | 64.51 | 15.26 | 36.52 |
| *Ca. ruelliae*_NRRL Y-48703 | 11134 | *Ca.* haemuli_NRRL Y-6693 | 11416 | 4819 | 76.68 | 12.92 | 43.28 |
| *Ca. ruelliae*_NRRL Y-48703 | 11134 | *Ca. auris*__b11220 | 10710 | 4583 | 75.01 | 13.63 | 42.79 |
| ***Ca. cisalpinoae*_UFMG-CM-Y6065** | 12794 | *Ca. ruelliae*_NRRL Y-48703 | 11134 | 4905 | 81.77 | 12.07 | 44.05 |
| ***Ca. cisalpinoae*_UFMG-CM-Y6066** | 12794 | *Ca. heveicola*_NRRL Y-48716 | 10482 | 4665 | 74.21 | 13.37 | 44.5 |
| ***Ca. cisalpinoae*_UFMG-CM-Y6067** | 12794 | *Ca. pseudohaemuli*_NRRL Y-48733 | 10441 | 4709 | 74.93 | 13.35 | 45.1 |
| ***Ca. cisalpinoae*_UFMG-CM-Y6068** | 12794 | *Ca. duobushaemuli*_NRRL Y-17802 | 10456 | 4681 | 74.85 | 13.29 | 44.77 |
| ***Ca. cisalpinoae*_UFMG-CM-Y6069** | 12794 | *Ca. vulturna*_CBS 14366 | 11303 | 4511 | 75.1 | 13.37 | 39.91 |
| ***Ca. cisalpinoae*_UFMG-CM-Y6070** | 12794 | *Os. tolerans*_NRRL Y-48705 | 10869 | 3970 | 64.29 | 15.32 | 36.53 |
| ***Ca. cisalpinoae*_UFMG-CM-Y6071** | 12794 | *Ca. haemuli*_NRRL Y-6693 | 11416 | 4809 | 75.62 | 13.12 | 42.13 |
| ***Ca. cisalpinoae*_UFMG-CM-Y6072** | 12794 | *Ca. auris*__b11220 | 10710 | 4604 | 73.89 | 13.76 | 42.99 |
| *Ca. konsanensis*_NRRL Y-63889 | 11136 | ***Ca. cisalpinoae*_UFMG-CM-Y6065** | 12794 | 4641 | 74.08 | 13.31 | 41.68 |
| *Ca. konsanensis*_NRRL Y-63889 | 11136 | *Ca. ruelliae*_NRRL Y-48703 | 11134 | 4680 | 74.91 | 13.27 | 42.03 |
| *Ca. konsanensis_*NRRL Y-63889 | 11136 | *Ca. heveicola_*NRRL Y-48716 | 10482 | 5307 | 93 | 7.35 | 50.63 |
| *Ca. konsanensis*_NRRL Y-63889 | 11136 | *Ca. pseudohaemuli*_NRRL Y-48733 | 10441 | 4932 | 83.69 | 11.12 | 47.24 |
| *Ca. konsanensis*_NRRL Y-63889 | 11136 | *Ca. duobushaemuli*_NRRL Y-17802 | 10456 | 4921 | 83.55 | 11.04 | 47.06 |
| *Ca. konsanensis*_NRRL Y-63889 | 11136 | *Ca. vulturna*_CBS 14366 | 11303 | 4730 | 83.63 | 11.07 | 42.47 |
| *Ca. konsanensis*_NRRL Y-63889 | 11136 | *Os. tolerans*_NRRL Y-48705 | 10869 | 3970 | 64.54 | 15.09 | 36.53 |
| *Ca. konsanensis*_NRRL Y-63889 | 11136 | *Ca. haemuli*_NRRL Y-6693 | 11416 | 4877 | 79.33 | 12.37 | 43.79 |
| *Ca. konsanensis*_NRRL Y-63889 | 11136 | *Ca*. *auris*__b11220 | 10710 | 4600 | 75.1 | 13.39 | 42.95 |
| *Os. _mogii*_NRRL Y-17032 | 7515 | *Ca. konsanensis*_NRRL Y-63889 | 11136 | 4351 | 64.4 | 14.96 | 57.9 |
| *Os. _mogii*_NRRL Y-17032 | 7515 | ***Ca. cisalpinoae*_UFMG-CM-Y6065** | 12794 | 4323 | 64.22 | 15.12 | 57.52 |
| *Os. _mogii*_NRRL Y-17032 | 7515 | *Ca. ruelliae*_NRRL Y-48703 | 11134 | 4351 | 64.5 | 15.03 | 57.9 |
| *Os. _mogii*_NRRL Y-17032 | 7515 | *Ca. heveicola*_NRRL Y-48716 | 10482 | 4390 | 64.54 | 14.94 | 58.42 |
| *Os. _mogii*_NRRL Y-17032 | 7515 | *Ca. pseudohaemuli*_NRRL Y-48733 | 10441 | 4403 | 64.72 | 15 | 58.59 |
| *Os. _mogii*_NRRL Y-17032 | 7515 | *Ca. duobushaemuli*_NRRL Y-17802 | 10456 | 4414 | 64.71 | 15 | 58.74 |
| *Os. _mogii*_NRRL Y-17032 | 7515 | *Ca. vulturna*_CBS 14366 | 11303 | 4221 | 65.06 | 15.08 | 56.17 |
| *Os. _mogii*_NRRL Y-17032 | 7515 | *Os. tolerans*_NRRL Y-48705 | 10869 | 4484 | 72.23 | 14.46 | 59.67 |
| *Os. _mogii*_NRRL Y-17032 | 7515 | *Ca*. *haemuli*_NRRL Y-6693 | 11416 | 4478 | 64.86 | 14.97 | 59.59 |
| osmozyma_mogii_NRRL Y-17032 | 7515 | *Ca. auris__*b11220 | 10710 | 4303 | 64.69 | 15.21 | 57.26 |
| *Ca. chanthaburiensis_*CBS 10926 | 11161 | *Os. mogii*_NRRL Y-17032 | 7515 | 4376 | 64.48 | 14.92 | 58.23 |
| *Ca. chanthaburiensis*_CBS 10926 | 11161 | *Ca. konsanensis*_NRRL Y-63889 | 11136 | 5953 | 96.18 | 6.52 | 53.46 |
| *Ca. chanthaburiensis*_CBS 10926 | 11161 | ***Ca. cisalpinoae*_UFMG-CM-Y6065** | 12794 | 4656 | 74.07 | 13.3 | 41.72 |
| *Ca. chanthaburiensis*_CBS 10926 | 11161 | *Ca. ruelliae*_NRRL Y-48703 | 11134 | 4698 | 74.92 | 13.21 | 42.2 |
| *Ca. chanthaburiensis*_CBS 10926 | 11161 | *Ca. heveicola*_NRRL Y-48716 | 10482 | 5336 | 93.13 | 6.9 | 50.91 |
| *Ca. chanthaburiensis*_CBS 10926 | 11161 | *Ca. pseudohaemuli*_NRRL Y-48733 | 10441 | 4955 | 83.77 | 10.9 | 47.46 |
| *Ca. chanthaburiensis*_CBS 10926 | 11161 | *Ca*. *duobushaemuli*_NRRL Y-17802 | 10456 | 4951 | 83.63 | 10.9 | 47.35 |
| *Ca. chanthaburiensis*_CBS 10926 | 11161 | *Ca*. *vulturna*_CBS 14366 | 11303 | 4753 | 83.62 | 11.01 | 42.59 |
| *Ca. chanthaburiensis*_CBS 10926 | 11161 | *Os*. *tolerans*_NRRL Y-48705 | 10869 | 4003 | 64.51 | 15.12 | 36.83 |
| *Ca. chanthaburiensis*_CBS 10926 | 11161 | *Ca*. *haemuli*_NRRL Y-6693 | 11416 | 4907 | 79.29 | 12.35 | 43.97 |
| *Ca. chanthaburiensis_*CBS 10926 | 11161 | *Ca*. *auris*__b11220 | 10710 | 4623 | 75.07 | 13.34 | 43.17 |

* Std: Standard deviation.

Table S4: Percentage of Conserved Proteins (POCP) among different *Candidozyma* species and other related species.

|  | *Candidozyma auris* B11220 | *Candidozyma chanthaburiensis* CBS 10926 | *Candidozyma haemuli* NRRL Y-6693 | *Candidozyma heveicola* NRRL Y-48716 | *Candidozyma konsanensis* NRRL Y-63889 | *Candidozyma pseudohaemuli* NRRL Y-48733 | *Candidozyma ruelliae* NRRL Y-48703 | *Candidozyma vulturna* CBS 14366 | *Candizoyma duobushaemuli*  NRRL Y-17802 |
| --- | --- | --- | --- | --- | --- | --- | --- | --- | --- |
| *Candidozyma cisalpinoae* UFMG-CM-Y6065 | 54.8 | 54.2 | 54.6 | 55.3 | 54.0 | 55.5 | 55.7 | 54.4 | 55.2 |

Table S5: Differential physiological characteristics among *Candidozyma cisalpinoae* sp. *nov*, and the other phylogenetically related *Candidozyma* species.

| Physiological attributes | *Ca. cisalpinoae* | *Ca. ruelliae* | *Ca. haemuli* | *Ca. auris* |
| --- | --- | --- | --- | --- |
| **Fermentation:** |  |  |  |  |
| Glucose | + | - | + | w/d |
| Maltose | w/d | - | - | - |
|  |  |  |  |  |
| **Assimilation:** |  |  |  |  |
| Cellobiose | + | + | - | - |
| L-Sorbose | + | + | - | - |
| L-Rhamnose | + | + | + | - |
| L-Arabinose | s | + | - | - |
| D-Ribose | - | + | d | w |
| Ethanol | s | + | d | - |
| Ribitol | - | + | d | w |
| Xylitol | s | + | w/d | ND |
| Succinate | + | + | + | - |
| Citrate | w/d | + | + | + |
|  |  |  |  |  |
| **Growth on YM agar at:** |  |  |  |  |
| 42 °C | w/s | w/s | - | w/s |
|  |  |  |  |  |
| **Other phenotypic tests:** |  |  |  |  |
| Tolerance to cycloheximide 0.01% (w/v) | + | + | + | - |
| Tolerance to cycloheximide 0.1% (w/v) | w/d | ND | ND | - |

All strains grow at temperatures of 25°C, 30°C and 35°C. The table was assembled according to the scoring system described by Kurtzman et al. (2011): +, positive; −, negative; d, late positive (latent); s, slowly positive; w, weakly positive; v, variable; ND, no data available.

Table S6:  MIC (Microdilution Inhibitory Concentration) for *Candida albicans* and each isolate of *Candidozyma cisalpinoae*.

| **MIC (μg/ml)** | |  |  |  |  |  |  |
| --- | --- | --- | --- | --- | --- | --- | --- |
| Strain | FLU | ITR | VOR | AMB | CAS | MCF | ANF |
| *C. albicans* SC5314 | 4 | 0.25 | 1 | 0.5 | 0.125 | 0.06 | 0.06 |
| UFMG-CM-Y6065 | 16 | 4 | 2 | 2 | 0.25 | 0.125 | 0.5 |
| UFMG-CM-Y6066 | 32 | 4 | 2 | 2 | 0.25 | 0.25 | 0.25 |
| UFMG-CM-Y7529 | 16 | 2 | 2 | 2 | 0.25 | 0.125 | 0.25 |
| UFMG-CM-Y7530 | 16 | 2 | 1 | 2 | 0.5 | 0.25 | 0.25 |
| UFMG-CM-Y7528 | 8 | 2 | 1 | 2 | 0.5 | 0.25 | 0.25 |
| UFMG-CM-Y7531 | 16 | 2 | 1 | 2 | 0.5 | 0.25 | 0.25 |

Minimal inhibitory concentration (MIC) to: Fluconazole (range of 0.125 to 64 µg/mL) (FLU), Itraconazole (range of 0.0313 to 16 µg/mL) (ITR), Voriconazole (VOR) (range of 0.0313 to 16 µg/mL), Amphotericin B (range of 0.0313 to 16 µg/mL) (AMB), Caspofungin (CAS), Micafungin (MCF), Anidulafungin (ANF) (range of 0.015 to 8 μg//mL).

Table S7: Adhesion of yeasts to oral epithelial cells for *Candida albicans* and each isolate of *Candidozyma cisalpinoae*.

| Strain | Total adhered yeast cells | Percentage of oral epithelial cells with adhered yeast |
| --- | --- | --- |
| *Candida albicans* SC5314 | 198 | 84% |
| *Candidozyma cisalpinoae* UFMG-CM-Y6065 | 139 | 80% |
| *Ca. cisalpinoae* UFMG-CM-Y6066 | 141 | 76% |
| *Ca. Cisalpinoae* UFMG-CM-Y7531 | 117 | 79% |
| *Ca. cisalpinoae* UFMG-CM-Y7530 | 107 | 82% |
| *Ca. cisalpinoae* UFMG-CM-Y7529 | 122 | 94% |
| *Ca. cisalpinoae* UFMG-CM-Y7528 | 102 | 74% |

Adhesion capacity to oral epithelial cells of selected isolates of *Candidozyma cisalpinoae* originating from flower environments in Tocantins - Brazil. *Candida albicans* SC5314 was used as positive control.

Table S8: Analysis of Biofilm formation (crystal violet absorbance) for *Candida albicans* and each isolate of *Candidozyma cisalpinoae*.

| **Strain** | **Biofilm** |
| --- | --- |
| *Candida albicans* SC5314 | 0.234 ± 0.799 |
| *Candidozyma cisalpinoae* UFMG-CM-Y6065 | 0.293 ± 0.803 |
| *Ca. cisalpinoae* UFMG-CM-Y6066 | 0.288 ± 0.850 |
| *Ca. cisalpinoae* UFMG-CM-Y7531 | 0.247 ± 0.687 |
| *Ca. cisalpinoae* UFMG-CM-Y7530 | 0.385 ± 0.889 |
| *Ca. cisalpinoae* UFMG-CM-Y7529 | 0.397 ± 0.855 |
| *Ca. cisalpinoae* UFMG-CM-Y7528 | 0.302 ± 0.708 |
